# Ovarian Control of Nectar Collection in the Honey Bee (*Apis mellifera*)

**DOI:** 10.1371/journal.pone.0033465

**Published:** 2012-04-30

**Authors:** Adam J. Siegel, Colin Freedman, Robert E. Page

**Affiliations:** 1 School of Life Sciences, Arizona State University, Tempe, Arizona, United States of America; 2 Department of Ecology, Evolution and Behavior, Alexander Silberman Institute of Life Sciences, The Hebrew University of Jerusalem, Jerusalem, Israel; 3 College of Liberal Arts and Sciences, Arizona State University, Tempe, Arizona, United States of America; University of Osnabrueck, Germany

## Abstract

Honey bees are a model system for the study of division of labor. Worker bees demonstrate a foraging division of labor (DOL) by biasing collection towards carbohydrates (nectar) or protein (pollen). The Reproductive ground-plan hypothesis of Amdam et al. proposes that foraging DOL is regulated by the networks that controlled foraging behavior during the reproductive life cycle of honey bee ancestors. Here we test a proposed mechanism through which the ovary of the facultatively sterile worker impacts foraging bias. The proposed mechanism suggests that the ovary has a regulatory effect on sucrose sensitivity, and sucrose sensitivity impacts nectar loading. We tested this mechanism by measuring worker ovary size (ovariole number), sucrose sensitivity, and sucrose solution load size collected from a rate-controlled artificial feeder. We found a significant interaction between ovariole number and sucrose sensitivity on sucrose solution load size when using low concentration nectar. This supports our proposed mechanism. As nectar and pollen loading are not independent, a mechanism impacting nectar load size would also impact pollen load size.

## Introduction

Task specialization and division of labor are principal features of insect societies and are believed to be the prime enablers of their ecological and evolutionary success [1]. Honey bees provide a model system for the study of task specialization and division of labor [2]–[4]. Reproduction is normally restricted to the queen and her male mates (drones). Facultatively sterile female workers perform all of the tasks associated with nest construction and maintenance, care of young, resource exploitation, and colony defense. Task performance by workers is age correlated; young workers perform in-hive tasks while older workers perform outside tasks. Typically, foraging outside the nest is performed by the oldest workers. Most honey bees specialize on carbohydrate or protein foraging by respectively biasing food gathering towards nectar (carbohydrate) or pollen (protein) collection [4]. The foraging behavior of thousands of workers results in a surplus of pollen and honey in the nest.

The Reproductive Ground-plan hypothesis (RGPH) is a framework for explaining the control of foraging division of labor. The RGPH suggests the regulatory mechanisms that controlled food collection during the reproductive life cycle of the solitary ancestor of the honey bee have been co-opted and modified to regulate foraging division of labor [5], [6]. The RGPH is one of the only well studied examples of this type of modification of a behavioral regulatory mechanism. Female solitary insects go through a reproductive life cycle, with a non-reproductive stage characterized by inactive ovaries and carbohydrate feeding, and a reproductive stage characterized by activated ovaries and protein feeding. In honey bees, ovary size (measured by counting ovarioles, the egg producing filaments of the ovary) is determined during larval development. Honey bee foragers with larger ovaries (more ovarioles), a reproductively associated characteristic, are biased toward protein collection compared to those with smaller ovaries (fewer ovarioles). This relationship between ovariole number and foraging preference has been demonstrated in honey bees selected for pollen storage levels as well as unselected wild-type *Apis mellifera* and *Apis cerana* foragers [2], [5]–[7]. According to the RGPH, there is a causal relationship between the worker ovary and foraging behavior.

Recent studies using workers derived from a backcross between European-Africanized Hybrid (EHB×AHB) queens and Africanized (AHB) drones further supported the RGPH by demonstrating that ovary size is associated with the individual foraging decisions of workers [8]. The (EHB×AHB)×AHB backcross studies demonstrated that ovary size and the sugar concentration of collected nectar have an impact on foraging bias. The impacts of these factors were not independent. Ovariole number and nectar concentration had an interaction effect on the proportion of the total foraging load that was pollen. This demonstrates that foragers with more ovarioles make different carbohydrate and protein loading decisions in response to the sugar concentration of nectar than do foragers with fewer ovarioles [8]. In addition to impacting food collection decisions, reproductive status has been shown to correlate with sugar response in many animal systems [9], [10]. Non-reproductive honey bee workers exhibit a similar relationship. Worker bees with more ovarioles are more sensitive to sucrose stimulation than worker bees with fewer ovarioles [11]. We hypothesize that the ovary regulates sensory sensitivity, which in turn affects nectar volume foraging decisions. We tested the hypothesis by investigating the relationship between ovariole number, sucrose sensitivity, and the amount of sucrose solution collected by honey bee workers foraging at a flow-rate controlled feeder. Using a flow-rate feeder, it was possible to determine sucrose collection volume without destroying the sampled bees. This allowed for the further collection of sucrose sensitivity data for each collected experimental forager. The flow-rate feeder also allowed for the approximation of natural nectar delivery under controlled conditions. This study was the first time that sucrose collection volume, sucrose sensitivity, and ovariole number have been measured for a set of bees under highly controlled conditions that still emulate natural floral nectar delivery.

## Results

### Experiment 1: Test of Time Spent on Feeder as an Estimate of Crop Load

**Figure 1:**
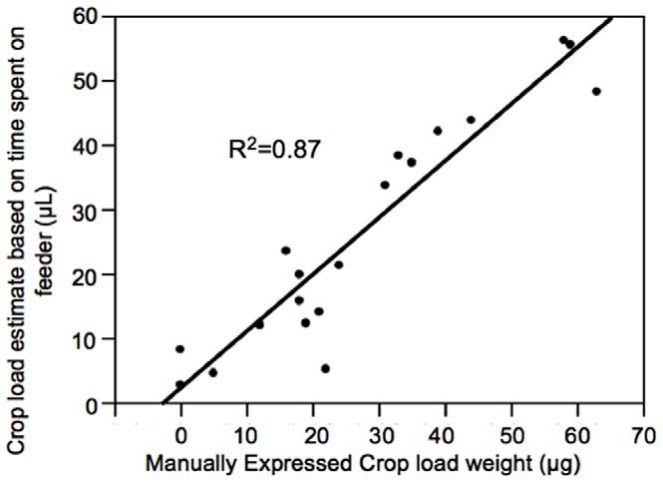
Linear relationship of crop load estimate (based on time spent on feeder) compared to manually expressed crop load weight.

There was a strong positive correlation between load size estimate based on time spent collecting from the rate-controlled feeder multiplied by solution flow rate and load size estimate based on manually expressing collected sucrose solution from the crop (Regression Analysis, F-ratio = 122.44, N = 19, P<0.0001). This relationship was linear (R^2^ = 0.89, Figure 1).

### Experiment 2: Test of Control for Previous Sucrose Concentration Exposure

**Figure 2:**
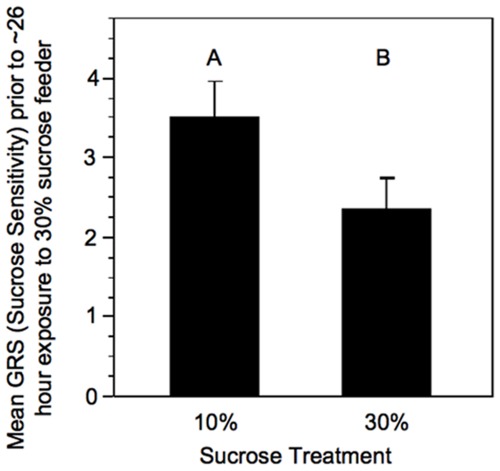
Mean (+SE) GRS (sucrose sensitivity) of bees after three days exposure to either 10% or 30% concentration sucrose solution. Letters signify significant difference (p<0.05).

**Figure 3:**
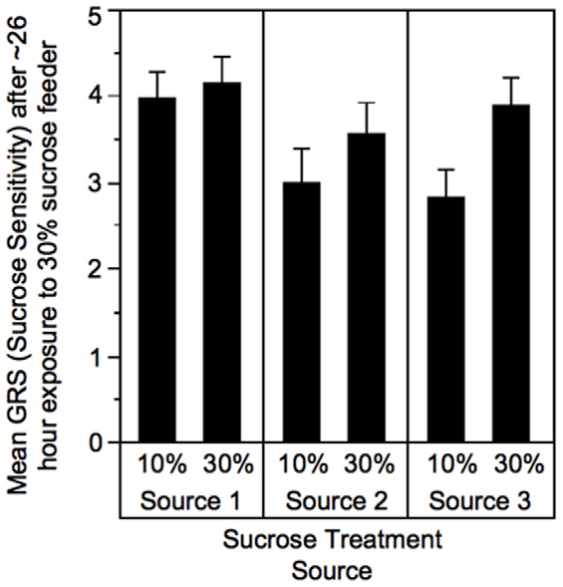
Mean (+SE) GRS (sucrose sensitivity) of bees after three days exposure to either 10% or 30% concentration sucrose solution followed by one day of additional exposure to 30% concentration sucrose solution. No significant differences in sensitivity were found regardless of original conditioning.

As expected, after three days exposure to differing concentrations of sucrose, the bees exposed to a 10% sucrose solution were significantly more responsive to sucrose than those exposed to the 30% sucrose solution (One-tailed Student’s t-test, t-ratio = −1.93, N_10%_ = 26, N_30%_ = 23, p<0.05, Figure 2, [12]). After all remaining bees had been given 24–29 hours access to an *ad lib* 30% sucrose feeder, there was no longer any difference in sucrose responsiveness between bees that had previously been exposed to 10% sucrose and those exposed to 30% sucrose for bees from any of the three sources, thus validating our methods (one-tailed Student’s t-test, t-ratios: Source 1 = 0.44, Source 2 = 1.12, Source 3 = 2.43, N = 32–45 for each group, p>0.05 for all sources, Figure 3).

### Experiment 3: Relationship between Ovariole Number, Sucrose Sensitivity, and Sucrose Collection

#### Differences between bees captured on 10% sucrose feeder and 30% sucrose feeder

**Figure 4:**
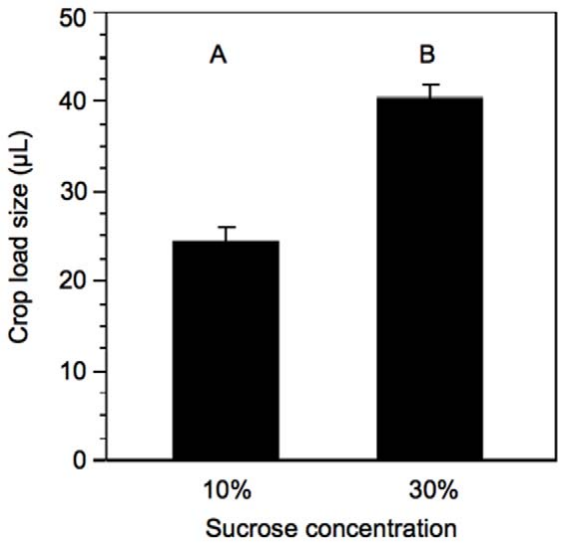
Mean (+SE) volume of sucrose collected by bees collected on 10% or 30% sucrose feeder. Letters signify significant difference (p<0.05).

**Figure 5:**
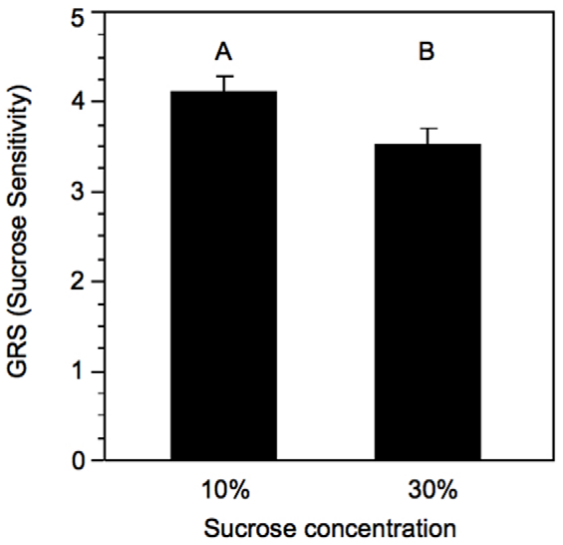
Mean (+SE) GRS (sucrose sensitivity) of bees collected on 10% or 30% sucrose feeder. Letters signify significant difference (p<0.05).

Honey bees captured on the 10% feeder collected significantly less sucrose solution than those captured on the 30% feeder (Student’s t-test, t-ratio = 7.70, N_10%_ = 155, N_30%_ = 158, p<0.0001, Figure 4). This is consistent with previous findings [13]. In addition, honey bees that accepted the 10% feeder were more sensitive to sucrose in lab assays, even after controlling for experience by allowing bees to feed on 30% sucrose for 26–29 hours prior to GRS testing (Student’s t-test, t-ratio = −2.32, N_10%_ = 131, N_30%_ = 142, p<0.005, Figure 5). This demonstrates that the bees accepted the feeders according to sucrose sensitivity.

#### Ovary size and sucrose sensitivity relationship with sucrose collection

Statistical analysis indicated a significant interaction effect between ovariole number and sucrose sensitivity on sucrose collection volume for bees foraging on 10% sucrose (GLMM, N = 131, Table 1). No other factors demonstrated an independent significant effect on sucrose collection volume, and there was no source colony effect. There were no significant effects on volume of 30% sucrose collected (Generalized Mixed Linear Model, N = 138, Table 2).

**Table 1:** Factors impacting volume of 10% sucrose solution collected treating GRS as an Ordinal Variable.

GLMM parameter estimates (Est.), standard errors (SE), and P values of potential factors IMPACTING 10% sucrose solution load size			
Parameter	Est.	SE	Statistical Significance
Intercept	25.05	5.54	****
Total Ovariole Number	−0.11	0.49	−
GRS	0.12	0.85	−
Total Ovariole Number × GRS	−0.49	0.23	*
Hive ID	2.69	2.33	−

Note that there is a significant interaction effect of ovariole number and GRS (sucrose sensitivity). Hive ID includes error caused by Colony source of bees and temporal pattern of data collection (Generalized Linear Mixed Model, N = 131, * = p<0.05, **** = p<.0001).

**Table 2:** Factors impacting volume of 30% sucrose solution collected.

GLMM parameter estimates (Est.), standard errors (SE), and P values of potential factors IMPACTING 30% sucrose solution load size			
Parameter	Est.	SE	P value
Intercept	45.32	4.49	****
Total Ovariole Number	−0.08	0.47	−
GRS	−0.71	0.70	−
Total Ovariole Number × GRS	−0.02	0.19	−
Hive ID	−0.48	2.29	−

* Hive ID includes error caused by colony source of bees and temporal pattern of data collection. Hive ID includes error caused by Colony source of bees and temporal pattern of data collection (Generalized Linear Mixed Model, N = 138, **** = p<.0001).

## Discussion

The results of this study demonstrate a link between ovariole number, sucrose sensitivity and nectar collection. These results support a proposed foraging division of labor control mechanism where the ovary impacts sucrose responsiveness in honey bees. Sucrose responsiveness, in turn, impacts the loading of sugar rich nectar. This mechanism fits well into the evolutionary RGPH that mechanisms controlling food collection during the life cycle of solitary ancestors of honey bees have been co-opted and remodeled to control foraging decisions in extant honey bees.

In this series of experiments, collected sucrose volume was estimated by multiplying the time foragers spent collecting sucrose solution from a delivery rate-controlled artificial feeder by the known solution flow rate [14]. The rate-controlled feeder had several benefits over an *ad lib* feeder. First, it more closely resembles natural conditions, as many insect pollinated flowers deliver nectar at extremely restricted rates [15]. Second, when exposed to the unnatural conditions of an *ad lib* feeder, honey bees are much less likely to make a discriminating foraging decision [16]. This is possibly due to the minimal foraging costs under these conditions. A forager can completely fill its crop in under 60 seconds on an *ad lib* feeder, compared to 15–20 minutes on natural flowers or rate-controlled feeders [13], [17].

We observed a strong linear relationship between the physically measured crop load size and the crop load estimate based on time spent on the rate-controlled feeder (Figure 1). This relationship validates the use of the time based estimate as a consistent non-destructive measure of foraging crop load size. As it is impossible to completely empty the crop of a forager by squeezing, the time based estimate may be a more accurate measure of crop load size than the standard squeezing technique. Additionally, bees imbibe all liquid in the feeder, further supporting the accuracy of this method.

We observed no difference in sucrose sensitivity between caged bees previously exposed to 10% sucrose and bees previously exposed to 30% sucrose, after one day of exposure of all bees to 30% sucrose feeders. From this, we conclude that one-day exposure to a common sucrose solution is sufficient to negate sucrose sensitivity effects of previous sucrose solution experience. Therefore, differences in sucrose sensitivity observed between bees collected on field feeders of different sucrose concentration after the one day cage treatment were due to the sorting of bees between sucrose feeders of differing sucrose concentration according to individual gustatory sensitivity. Bees that were more sensitive to sucrose accepted the 10% solution and the 30% solution; those that were less sensitive accepted only the 30% solution.

Bees collected larger loads of 30% sucrose solution than 10% sucrose solution (Figure 4). This demonstrates that bees are able to assess the relative value of nectar. Recently, Mujagic et al. [16] found no difference in time spent by foragers collecting sucrose solution (which can be used as a measure of collection volume- see methods) of different sucrose concentrations. However, the differences between their results and ours may be explained by their use of an *ad lib* feeder. Increased flow rate is positively correlated with crop load size [13]. Honey bees are able to completely fill their crops in fewer than 60 seconds when exposed to an *ad lib* feeder. This removes much of the cost associated with increased time spent foraging, and likely masks effects of different concentrations of sucrose solutions.

Bees collected on the 10% feeder demonstrated higher average sucrose sensitivity than bees collected on the 30% feeder, even after one day exposure to 30% sucrose feeders (Figure 5). The results of this study again differ from those of Mujagic et al (2010). They failed to demonstrate a relationship between sucrose sensitivity and acceptance thresholds of free flying bees. However, again methodological differences probably explain the differences in results. Mujagic et al. [18] used *ad lib* feeders to determine the field acceptance threshold of bees. Our study used flow-rate limited feeders. Because increased sugar concentration and increased solution flow rate both positively impact solution collection [13], it is likely that many bees in their study collected solutions in the field of a lower sucrose concentration than they would accept under the more natural conditions of restricted sucrose solution delivery, masking any effects of sucrose sensitivity on acceptance of sugar solution. Additionally, previous experience impacts sucrose sensitivity [12]. Testing bees without a control for experience would also mask differences in sucrose response sensitivity.

**Figure 6:**
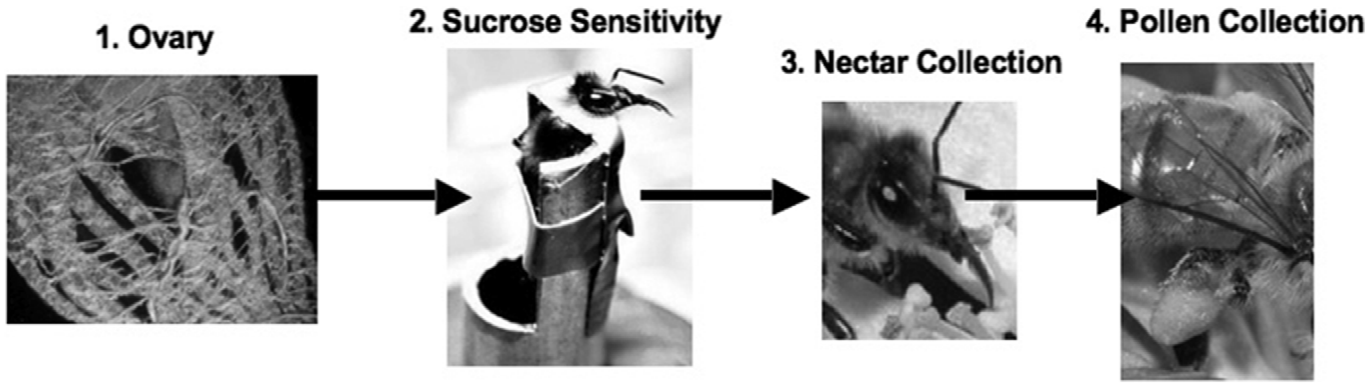
Proposed mechanism for ovary impact of foraging bias. The ovary (1) tunes sucrose sensitivity (2), which impacts nectar collection decisions (3). This would indirectly impact pollen collection (4) due to physical limitations on collection quantity (Photos: 1. O. Kaftanoflu; 2. J. S. Engen; 3.&4. Z. Huang).

The results of this study support our hypothesis that the ovary modulates sucrose perception, which in turn affects the volume of nectar collected. An interaction effect between ovariole number and sucrose sensitivity on volume of solution collected was observed within the 10% sucrose group (Table 1), as would be expected if ovary is affecting gustatory response to sugar and gustatory sensitivity is impacting nectar collection. Bees with different numbers of ovarioles demonstrated different responses to sucrose concentration and this is impacting their foraging decisions regarding nectar loading. Nectar and pollen collection are not independent due to physical collection limitations (carrying more of one floral product necessitates carrying less of the other; [19]. Therefore, a nectar collection regulatory system should also indirectly impact pollen collection (Figure 6). Complementary work should focus on what specific physiological or hormonal components of the ovary influence sucrose perception. The interaction between ovary and sucrose perception was not observed in the 30% sucrose group. Thirty percent sucrose is a highly valuable resource even in unrestricted environments. The majority of bees captured on the 30% sucrose feeder had near maximum foraging load sizes. We believe that the response to high sucrose concentration in a resource limited environment masked any potential foraging decisions due to ovary size.

### Conclusions

This study elucidates a mechanism regulating foraging division of labor that links ovariole number with sucrose sensitivity, and nectar loading decisions. As nectar loading and pollen loading are coupled due to physical loading constraints, a mechanism impacting nectar loading would also impact pollen loading. The results of this study demonstrate a link between reproductively associated phenotypes and foraging behavior in non-reproductive honey bee workers. This supports the RGPH, that reproductively associated regulation has been co-opted and reshaped to impact foraging division of labor. This sheds light on the transition from solitary to social behavior in Hymenoptera.

## Materials and Methods

In this series of experiments, the relationship between ovariole number, sucrose sensitivity, and sucrose collection was investigated in wild-type bees. The experiment was designed to test the hypothesis that ovariole number has a modulating effect on sucrose perception, which in turn impacts nectar collection. The experiments were conducted October–November, 2009 at the Arizona State University Bee Facility in Mesa, AZ. Three non-simultaneous replicates were performed using 10% and 30% sucrose solutions. Prior to beginning the main experiment (Experiment 3), we confirmed that time on an artificial feeder was an accurate method for estimating collected sucrose volume (Experiment 1). We also confirmed that it was possible to control for the effects of previous foraging experience on sucrose sensitivity (Experiment 2).

### Experiment 1: Test of Time Spent on Feeder as an Estimate of Crop Load

We used a method developed by Núñez (1971) to estimate crop load, where time spent imbibing from a sucrose solution delivery rate-controlled artificial feeder is multiplied by solution flow rate. The rate-controlled feeder has been suggested as a non-destructive method for measuring collected sucrose volume [14], [17]. Established methods of crop load estimation involve physically expressing crop contents, a technique that can damage or kill study animals. To test the accuracy of the proposed rate-controlled feeder method of crop load estimation, we timed a group of bees while they collected from the rate-controlled feeder and then expressed and weighed their crop loads using the traditional method. If time spent imbibing from the rate-controlled feeder multiplied by flow rate is an accurate index for measuring crop load size, there should be a significant linear relationship between crop load estimate based on time spent collecting and physically expressed crop load size.

A rate-controlled feeder containing 30% sucrose solution was set up at the Arizona State University Apiary in Mesa, AZ. A population of foragers from several nearby wild-type colonies of commercial origin was established at the feeder. Tested foragers could have been from any of more than a dozen nearby colonies. Twenty bees were timed while collecting solution, and then each bee was collected and narcotized using carbon dioxide. The crop load was expressed into a capillary tube by manually squeezing the abdomen, then weighed. One bee ruptured during this process and was excluded from analysis. A regression analysis was used to compare estimated crop load volume (time spend imbibing from the feeder multiplied by flow rate) to crop load weight determined by manually expressing collected solution.

### Experiment 2: Test of Control for Previous Sucrose Concentration Exposure

Honey bees demonstrate a baseline sucrose sensitivity that can be modulated by experience [12], [20], [21]. In experiment 3, bees were given access to feeders containing either a 10% sucrose solution feeder or a 30% sucrose solution feeder (only one feeder was present at a time). We wanted to determine the baseline sensitivity of bees captured on the two feeders, as baseline sucrose sensitivity is believed to affect the collection decisions of bees on the different feeders. However, experience at the feeders modulates the sucrose sensitivity response, which could mask our ability to measure the baseline sensitivity [20]. Therefore, we exposed collected bees to a common feeding environment prior to measuring sucrose sensitivity to control for experience on the feeder.

Three-hundred newly emerged wild-type honey bee workers from each of three wild-type sources (900 total) were paint marked (Testors Enamel) on the thorax and abdomen over a three day period and split evenly between two wild-type background colonies. A unique color combination was used for each source on each day. After bees had been in the colonies for 10 days, all marked bees observed outside the hive entrance over a three hour period in the morning were captured at the hive entrance and discarded to allow for maximum control of the food collection experience of experimental bees. The remaining marked bees were collected from the inside of the hives, randomly divided into groups of twenty and placed into small wire cages (∼10×10×20 cm). Half of the cages had 10% *ad libitum* sucrose solution feeders installed. The remainder had 30% *ad lib* sucrose solution feeders installed. Ten days after introduction was chosen for collection to give the bees time to mature in the colony, but allow for collection of the majority of the marked bees prior to foraging initiation. The cages were kept in an incubator (35°C, 50% RH) for 3 days, after which, a random subset of 30 bees of mixed origin was collected across cages for each concentration. Sucrose responsiveness was determined for the subset of bees exposed to 10% and 30% sucrose using a proboscis extension response (PER) assay to generate a gustatory response score (GRS; [22]–[24]).

**Figure 7:**
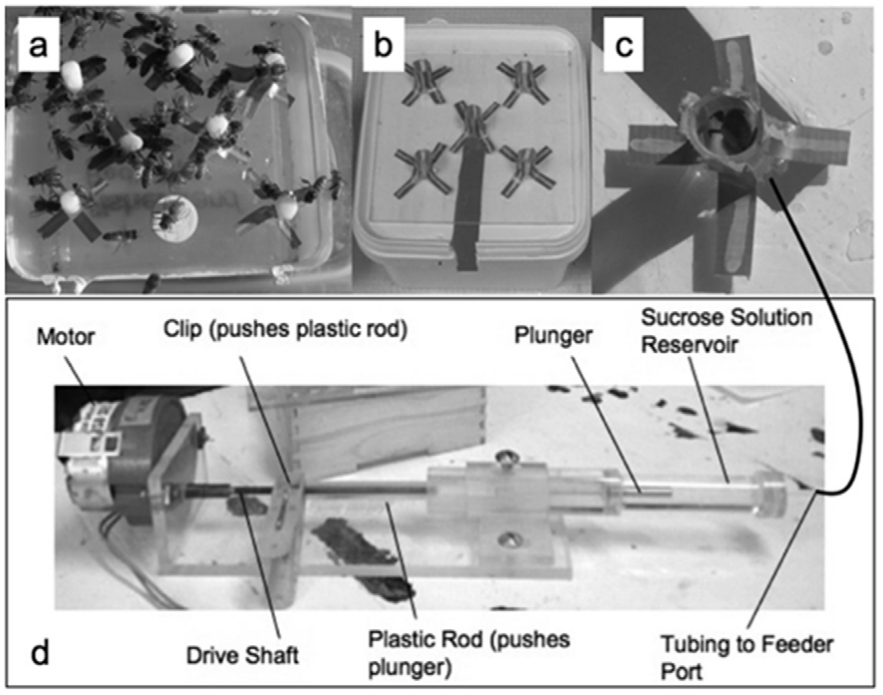
Sucrose feeders. (a) *Ad libitum filter* feeder. (b) Transitional *ad lib* tube feeder. (c) Honey bee forager inside rate restricted feeder port. (d) Rate restricted sucrose.

Bees were cooled to 4°C until immobile and then individually restrained in small tubes. Restrained bees were allowed to acclimate to the experimental conditions in an incubator (35°C, 50% RH) for at least 60 minutes. After the acclimation period, bees were allowed to drink water *ad lib* to avoid false positive responses due to dehydration [12], [25], [26]. Bees were then tested by stimulating both antennae with an ascending logarithmic sucrose concentration series (0, 0.1, 0.3, 1, 3, 10, 30% sucrose by weight) and honey. An inter-trial interval of at least 3 minutes was maintained. The GRS was determined by counting the number of concentrations for which a bee extended her proboscis in response to the antennal stimulation. Honey was included to test for physical PER ability of experimental bees. Occasionally the proboscis of a bee will become stuck below the rim of the restraining tube. Bees will generally demonstrate a PER to honey unless they are physically restrained from doing so. The “honey test” avoids including bees in the analysis that demonstrate false negative responses due to accidental physical constraint. Bees that did not respond to honey were excluded from the experiment. GRS for the 10% and 30% exposed bees was compared using a one-tailed Student’s t-test. A one-tailed test was used because of the *a priori* expectation that bees exposed to 10% sucrose would be more responsive than bees exposed to 30% sucrose. The tested subset of bees was then discarded.

To determine if honey bee sucrose responsiveness could be quickly reconditioned, all cages then had the *ad lib* feeders replaced with 30% sucrose *ad lib* feeders. After 24–29 hour exposure to the 30% sucrose feeders a GRS was determined for all remaining bees. The GRS of the bees that had been exposed to three days 10% sucrose solution followed by one day of 30% sucrose solution was then compared to the GRS of the bees that had been exposed to three days of 30% sucrose solution followed by an additional day of 30% sucrose solution, separately for bees from each original source.

### Experiment 3: Relationship between Ovariole Number, Sucrose Sensitivity, and Sucrose Collection

Several wild-type colonies were screened for worker ovariole number. Three source colonies were chosen that demonstrated high variation in ovariole number across workers (Colony 1: range: 0–52, mean: 7.0; Colony 2: range: 2–18, mean: 8.1; Colony 3: range: 1–14, mean: 6.6). Colony strength was estimated at over 10,000 workers for all chosen colonies. All experienced foragers were removed from the source colony prior to the initiation of data collection [27]. Colonies were placed in outdoor 6×12 m screen flight cages 2–4 days prior to starting data collection. Using the flight cage allowed for complete control over available foraging resources.

Once a new foraging population of several hundred workers was re-established, foragers were trained over 1 day to collect either 10% or 30% sucrose solution from *ad lib* artificial flower feeders 6 m from the entrance of the hive (Figure 7a). Only one concentration was available at a time. When a population of foragers was established at the pre-established collection site, the feeder was replaced with a visually similar *ad lib* feeder that required the bees to crawl into a small tube to access the sucrose reward (Figure 7b). When bees had learned to navigate the tube feeder, the feeder was replaced again with a flow rate-controlled feeder set at a solution delivery rate of 3.73 µl/min, [14], Figure 7c–d).

Crop load size based on time at the feeder was estimated for 50–53 bees captured on the feeder for each concentration and replicate over a period of 4–6 days. Prior to testing, the feeder was allowed to run for 60 seconds to build up a small reservoir of sucrose solution to attract foragers. This volume was included in the collection volume estimate. When a single bee entered the feeder port, time collection was initiated and a small wire cage (3×3×12 m) was placed over the opening to exclude other bees from the port. The cage avoided competition effects. As honey bees will often stop and start collection, the bee was allowed to continue collection until it had ceased collection for 60 continuous seconds. At this time, the focal bee was captured in the small wire cage. The time spent on the feeder plus the initial 60 second ‘charge’ was multiplied by the flow rate of 3.73 µL/min to estimate crop load volume.

At the end of each day’s collection period, all captured foragers were individually paint-marked (Testors Enamel) and split between two large wire cages with access to 30% sucrose *ad lib* feeders and kept for 26–29 hours in an incubator (35°C, 50% RH). This sequestration was performed to control for sucrose exposure experience so that we could compare sucrose sensitivity of bees collected on feeders containing different sucrose concentrations. Sucrose responsiveness was determined after 26–29 hours in the incubator by generating a GRS using the protocol outlined above. After the behavioral assays, the bees were dissected under magnification and ovarioles (egg producing filaments) were counted for both ovaries as an index of ovary size.

Student’s t-tests were conducted to compare the sucrose solution volume collected at 10% vs. 30% sucrose and to compare the GRS of bees collected on the 10% feeders vs. the 30% feeders. Source colony replicates were pooled for the volume and GRS comparisons, as source colony had no effect on collection volume (see results). A Generalized Linear Mixed Model (GLMM; JMP) was constructed to determine which factors impacted the volume of collected sucrose. Total Ovariole number and GRS were set as fixed factors. Hive ID (source colony) was set as a random factor. Bees for each concentration were analyzed separately. The model included ovariole number, GRS (sucrose sensitivity), ovariole number*GRS interaction and Hive ID as the error factor. Because the three replicates were conducted sequentially, Hive I.D. includes noise due to the temporal order of the replicates, colony source of the bees, or any additional potential replicate impact (i.e. genotype of the bees, quantity of brood in the hive, etc.).
